# Supplementary material for: Multi‐dataset identification of innovative feature genes and molecular mechanisms in keratoconus
Source: J Cell Mol Med. 2024 Sep 19;28(18):e70079. doi: 10.1111/jcmm.70079 (PMC11412914; doi:10.1111/jcmm.70079)
Supplement: Supplementary file 2 — Material S2. [file JCMM-28-e70079-s002.zip › 7.GSEA analysis/6.SNORA74B/KEGG_NITROGEN_METABOLISM.html]

Details for gene set KEGG\_NITROGEN\_METABOLISM[GSEA]

|  || Dataset | SNORA74B.group.cls#h\_versus\_l |
| Phenotype | group.cls#h\_versus\_l |
| Upregulated in class | l |
| GeneSet | KEGG\_NITROGEN\_METABOLISM |
| Enrichment Score (ES) | -0.5499077 |
| Normalized Enrichment Score (NES) | -1.4149135 |
| Nominal p-value | 0.06603774 |
| FDR q-value | 0.32750764 |
| FWER p-Value | 0.884 |
Table: GSEA Results Summary

  

Fig 1: Enrichment plot: KEGG\_NITROGEN\_METABOLISM      
 Profile of the Running ES Score & Positions of GeneSet Members on the Rank Ordered List

  

| SYMBOL | TITLE | RANK IN GENE LIST | RANK METRIC SCORE | RUNNING ES | CORE ENRICHMENT || 1 | CA5A | na | 3753 | 0.269 | -0.0420 | No |
| 2 | GLUD1 | na | 10973 | 0.075 | -0.2314 | No |
| 3 | CA5B | na | 11075 | 0.074 | -0.2162 | No |
| 4 | CA7 | na | 11729 | 0.064 | -0.2194 | No |
| 5 | CA9 | na | 18112 | -0.007 | -0.4015 | No |
| 6 | GLUD2 | na | 19722 | -0.026 | -0.4416 | No |
| 7 | GLUL | na | 20107 | -0.030 | -0.4454 | No |
| 8 | GLS | na | 20289 | -0.031 | -0.4429 | No |
| 9 | CA13 | na | 21768 | -0.049 | -0.4734 | No |
| 10 | CA3 | na | 23089 | -0.067 | -0.4950 | No |
| 11 | ASNS | na | 24265 | -0.085 | -0.5080 | No |
| 12 | CA6 | na | 25561 | -0.109 | -0.5186 | Yes |
| 13 | AMT | na | 26650 | -0.131 | -0.5176 | Yes |
| 14 | CA4 | na | 27063 | -0.141 | -0.4948 | Yes |
| 15 | GLS2 | na | 27487 | -0.151 | -0.4699 | Yes |
| 16 | CPS1 | na | 27845 | -0.161 | -0.4407 | Yes |
| 17 | HAL | na | 29309 | -0.204 | -0.4327 | Yes |
| 18 | CA14 | na | 30855 | -0.260 | -0.4131 | Yes |
| 19 | CTH | na | 31622 | -0.302 | -0.3609 | Yes |
| 20 | CA8 | na | 32131 | -0.330 | -0.2944 | Yes |
| 21 | CA1 | na | 33223 | -0.415 | -0.2236 | Yes |
| 22 | CA12 | na | 33884 | -0.509 | -0.1175 | Yes |
| 23 | CA2 | na | 34181 | -0.576 | 0.0158 | Yes |
Table: GSEA details [plain text format]

  

Fig 2: KEGG\_NITROGEN\_METABOLISM      
 Blue-Pink O' Gram in the Space of the Analyzed GeneSet

  

Fig 3: KEGG\_NITROGEN\_METABOLISM: Random ES distribution      
 Gene set null distribution of ES for **KEGG\_NITROGEN\_METABOLISM**
